# Nutrition Transition with Accelerating Urbanization? Empirical Evidence from Rural China

**DOI:** 10.3390/nu13030921

**Published:** 2021-03-12

**Authors:** Yanjun Ren, Bente Castro Campos, Yanling Peng, Thomas Glauben

**Affiliations:** 1 Department of Agricultural Markets, Leibniz Institute of Agricultural Development in Transition Economies (IAMO), 06120 Halle, Germany; ren@iamo.de (Y.R.); glauben@iamo.de (T.G.); 2 Department of Agricultural Policy and Market Research, Faculty of Agricultural Sciences, Nutritional Sciences, and Environmental Management, Justus Liebig University Giessen, 35390 Giessen, Germany; 3 Institute of Agricultural Economics, Faculty of Agricultural and Nutritional Sciences, Kiel University, 24118 Kiel, Germany; 4 College of Economics, Sichuan Agricultural University, Wenjiang District, Chengdu 611130, China; jxyanling@163.com

**Keywords:** urbanization, dietary transition, calorie intake, dietary quality, rural China

## Abstract

Although rapid urbanization is often considered as one of the most important drivers for changing dietary patterns, little attention has been paid to rural areas despite the profound transformation they have undergone. Using longitudinal data from the China Health and Nutrition Survey (CHNS) for the period from 2004 to 2011, this study seeks to better understand the relationship between the urbanization of rural areas and dietary transition, with the focus on nutrition intake and dietary quality. Our results suggest that with increasing urbanization, rural residents tend to have on average lower calorie intakes but higher dietary quality. Specifically, increasing urbanization consistently reduces carbohydrate consumption and reduces fat consumption after a turning point; protein consumption first decreases and then increases after the turning point with increasing urbanization. Urbanization shows a significant and positive effect on the Healthy Eating Index (HEI). In addition to sociodemographic changes, we find that changing consumer preferences and knowledge serve as important determinants in explaining the dietary transition in rural China from 2004 to 2011. In our study, urbanization appears to positively affect rural residents’ healthy food preferences and dietary knowledge. This study is a first attempt for better understanding the nutrition transition resulting from accelerating urbanization in rural China; several limitations and areas for future research have been highlighted.

## 1. Introduction

Dietary patterns have undergone profound changes worldwide, especially in a transition economy such as China in which consumers are switching from traditional Chinese food, which is largely characterized by grains and vegetables, to foods that are high in fat and protein [1,2]. In rural compared to urban China, incomes are lower and infrastructure and access to services, markets, and value chains are often limited [1,3,4,5]. As a result, there are still approximately 150.8 million people who are undernourished or suffer from micronutrient deficiencies in China, although overnutrition, such as overweight and obesity, has become a major public health issue [3,6,7].

Rapid urbanization globally is often considered as one of the most important drivers for changing dietary patterns and nutrition [5]. Higher urbanization, usually a characteristic of developed economies, can influence food consumption and nutrition transition in several ways. For instance, urbanization can affect consumption patterns through its effect on agricultural land, food supply and prices, and the food environment [8]. The food environment in highly urbanized regions is generally more diverse [9]. In developing economies, the ongoing expansion of supermarkets and fast food chains is still mainly concentrated in regions with greater urbanization [10]. This is expected to improve the food availability and food diversity of residents; however, individuals also have a higher likelihood of consuming processed or prepared food, or to dine out; these factors are often related to a poor dietary quality, such as through higher intakes of energy and sodium [11,12]. Although there is no universal definition for a healthy diet as different people in countries and age groups have largely different nutrition requirements [13], one consensus is achieved that a healthy diet should be in balance with energy expenditure [14]. Less healthful food consumption patterns are sometimes argued to be a result of urban lifestyles linked to the consumption of higher amounts of fast food and less physical activity [15]. An increasing number of people are employed in white collar jobs with lower calorie requirements [16], higher opportunity costs of time spent on acquiring and preparing food, and higher preference for more conveniently consumed and pre-prepared food [9,16,17]. The manner in which urbanization affects the intake of nutrients and dietary quality, and the extent of this impact, require further attention in the academic literature.

Although high-income countries have been highly urbanized for several decades, developing countries are now also rapidly urbanizing [5]. This is especially true for China, which has experienced significant urbanization in recent decades. As a result, urbanization in China increased from 17.9% in 1978 to approximately 59.6% in 2018, according to data from the China National Bureau of Statistics (www.stats.gov.cn/tjsj/zxfb/201908/t20190815_1691416.html, accessed on 11 November 2020). However, rural–urban inequality has increased due to urbanization, especially in food consumption and dietary patterns. Compared to urban residents, the nutritional intake from animal products remains low for rural residents [1,18,19]. Increasing urbanization is expected to drive significant changes in nutrition and dietary patterns in rural China toward Western consumption patterns. Especially, accelerated urbanization also enables rural residents to have a higher availability of diverse food commodities through an increasing number of supermarkets and the presence of online shopping [20,21].

Many studies have qualitatively or quantitatively discussed the effect of urbanization on the patterns of food consumption and nutrients in developing societies [5,22,23]. In Africa, it has been found that rural residents who migrate to urban areas tend to change their food consumption patterns considerably, for example, by decreasing their consumption of staple foods such as maize and increasing their consumption of processed food products and high-sugar foods [5]. Similar results have been revealed for China. After estimating the urbanization elasticities, it has been demonstrated that urbanization in China has increased the demand for meats, fruits, and eggs, while reducing the demand for grains, vegetables, and fats and oils [22]. After calculating the three-day average percentage of fat, it was found that increasing urbanization is associated with increasing fat in the diet. However, no further analysis of the other two macronutrients, proteins and carbohydrates, has been provided to date [23].

Furthermore, it has been debated that nutritional intake can only partially reveal the structure of diets [24]. A more comprehensive measure should be considered to examine a diet’s quality, such as the Healthy Eating Index (HEI), which is regarded as a good proxy of the nutrient adequacy of a diet [25,26,27]. Therefore, this study aims to fill this research gap by estimating the effect of urbanization on nutrition intake and dietary quality.

Generally, the two main mechanisms through which urbanization could have an effect on diet are through an economic and a non-economic channel. Regarding the economic channel, extensive studies have well documented how urbanization affects regional development and household or individual income, which in turn affect household or individual diets [4,8,22,28]. However, little is known about the non-economic channels, such as food preference and dietary knowledge, which serve as two potential channels through which urbanization might affect nutrition transition. Due to increasing urbanization, consumers’ preference for food consumption has largely shifted away from high-carbohydrate food toward high-energy food such as high fat and sugar-added food products [29,30,31,32], especially in transitional economies such as China. Dietary knowledge is considered another channel to explain the effect of urbanization on dietary transition. We expect that a strong link exists between urbanization and dietary knowledge because economic or regional development and a changing food environment help individuals easily obtain more sources of information concerning their diets and nutrition-related health behaviors [30,33].

Although a number of studies have discussed the impact of urbanization on food consumption, sound empirical and quantitative evidence is very limited. Among this limited previous research, one important study provides a detailed descriptive analysis of the relationship between urbanicity and the Chinese diet using data from the China Health and Nutrition Survey (CHNS) [34]; however, the nutrition changes for rural residents are not considered in their study. Despite the profound transformation that is taking place in rural areas, we know relatively little about dietary transition in rural China. To the best of our knowledge, no study has been conducted that focuses on the effect of urbanization on dietary transition in rural China. This paper extends the literature by studying the relationship between urbanization and dietary transition, using longitudinal data from the CHNS.

The main contributions of this study are threefold. First, diets are comprehensively measured using nutrition intake and dietary quality to shed light on the effect of urbanization on dietary transition. Second, we also investigate how urbanization affects food preference and knowledge. Third, considering the panel structure of our data, a pseudo-fixed-effects model is applied to address the individual heterogeneity in our estimations.

The remainder of this article is organized as follows. In Section 2, we present the materials and econometric method used in our estimation. Section 3 presents the main results. Section 4 discusses the implications and limitations of our empirical results. In Section 5, we draw conclusions.

## 2. Materials and Methods

### 2.1. Data

The CHNS data used in this study cover the period from 1989 to 2015. A multistage, random cluster design in eight provinces (Liaoning, Jiangsu, Shandong, Henan, Hubei, Hunan, Guangxi, and Guizhou) is used to select a stratified probability sample. The basis for this sample is the original survey of 1989. The provinces and municipalities differ considerably in their geography, economic development, public resources, and health indicators. Following the sampling strategy, the CHNS selects two cities (one large and one small, usually the provincial capital and a lower-income city) and four counties (stratified by income, one high-, one low-, and two middle-income) per province. Within cities, the CHNS selects two urban and two suburban communities. Within counties, the CHNS randomly assigns one community in the capital city and three rural villages. Within the communities and villages, twenty households per community were randomly selected for participation [35]. In doing so, a multistage and random cluster process is used to draw a sample of approximately 4400 households comprising approximately 26,000 individuals. With the 2011 survey, in addition to the eight provinces, the CHNS also contains Heilongjiang province and three autonomous cities (Beijing, Shanghai, and Chongqing), comprising 288 communities with 27,447 individuals from 5884 households. For the analysis of this article, we applied three restrictions to the CHNS dataset. First, information on macronutrients and consumer preference and knowledge are only available from 2004 to 2011; therefore, we used the data for 2004, 2006, 2009, and 2011. Second, we restricted the sample to adults living in rural areas and considered individuals aged 18–65 at the time of the survey. Overall, our sample includes 17,315 observations for 8023 individuals. The panel data are unbalanced.

### 2.2. Variables

The main dependent variables of this study are nutrition intake and dietary quality. To measure the nutrition intake, the CHNS provides information on dietary patterns for three consecutive days, in addition to information on nutritional content of these food items provided by the Chinese Food Nutrition Table 2002 [36]. To assess the detailed information for dietary patterns, the CHNS combined three consecutive 24-h recalls at the individual level and a food inventory taken at the household level over the same three-day period. The three-day average intakes of calories and the three macronutrients were calculated, including total calories (kcal), carbohydrates (g), fats (g), and proteins (g) consumed at the individual level.

The descriptive statistics of the main variables are presented in Table 1. The three-day average calorie intake is 2215.50 kcal with a standard deviation of 685.92, which is very close to the recommendation of the China Nutrition Society [37]. Nutritional intakes of macronutrients in our sample are comparatively larger than those from previous studies [38]. A comparison by gender indicates that females consumed 2029.46 kcal calories per day, which is significantly lower than the value of 2412.08 kcal for males.

**Figure 1:**
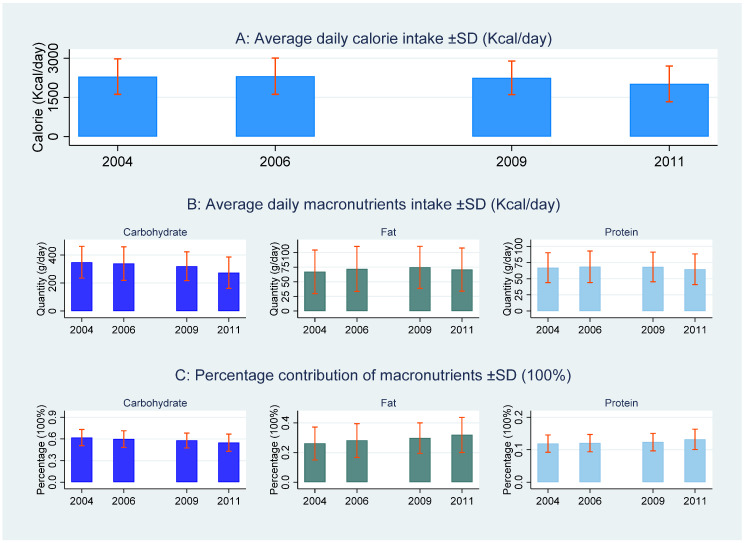
The trend of total calorie intakes and macronutrients from 2004 to 2011.

The percentage contribution of macronutrients to the total calories is calculated by the following standard: 1 g carbohydrate equals 4 kcal; 1 g fat equals 9 kcal; 1 g protein equals 4 kcal [39]. As shown in Table 1, the average intake of carbohydrates is 319.58 g and accounts for approximately 59% of total calories, indicating that carbohydrates are the main source of nutrition for rural residents. The intakes of fat and protein are 71.11 and 67.02 g, accounting for 29% and 12% of the total calorie intakes, respectively. As shown in Figure 1, the calorie and carbohydrate intakes (and their percentage contributions to calorie intake) show a decreasing trend from 2004 to 2011, while fat and protein intakes (and their percentage contributions to calorie intake) present a slightly increasing trend from 2004 to 2011. A possible reason for this observation is that the calorie increase from increased fat and protein intakes is lower than the calorie decrease from reduced carbohydrate intake. The comparison between males and females indicates that males have a greater intake of calories and the three macronutrients with the differences being statistically significant based on evidence from *t*-tests (Table 1).

Dietary quality was measured using the Healthy Eating Index (HEI) for Chinese, which was recently developed by Yuan et al. [40]. The HEI is based on the updated Dietary Guidelines for Chinese (DGC) in 2016 [37]. The HEI for Chinese was designed as a scoring system to assess dietary quality, considering 17 components in line with key dietary recommendations of the DGC. The HEI assesses dietary quality from two perspectives: 12 food groups evaluate the adequacy of a diet, namely, total grains, whole grains and mixed beans, tubers, total vegetables, dark vegetables, fruits, dairy, soybeans, fish and seafood, poultry, eggs, and seeds and nuts. The other five components assess dietary components that are recommended to be consumed in moderation, namely, red meat, cooking oil, sodium, added sugar, and alcohol. Thus, higher intakes of components of the first 12 groups will receive higher scores, whereas higher intakes of the last five components will receive lower scores. Therefore, higher total scores reflect better dietary quality. Considering all 17 components, a total score of the HEI ranges from 0 to 100. More details regarding the establishment of the HEI according to the quantity of food consumed are provided in the Supplementary Table S1. Since there is no information regarding added sugars in the CHNS, the maximum total score was 95 in this study. As shown in Table 1, the mean value of the HEI was 60.33; this is similar to the findings of Yuan et al. [40]. The HEI is significantly different between females and males, suggesting that females have a higher HEI than males.

The key explanatory variable urbanization (urbanicity) is measured by a comprehensive index [23]. The index covers 12 aspects of urbanization, namely, population density, economic activity, traditional markets, modern markets, transportation infrastructure, sanitation, communication, housing, education, diversity, health infrastructure, and social services. This index is designed to capture a community’s physical, social, cultural, and economic environments [23], and it has been widely used by other studies [28,34,41,42]. Regarding the construction of the index, each of the 12 components are assigned a value within a range of 0–10; thus, the overall maximum score of the index is 120. The larger the index, the higher the urbanization level of the region.

Regarding the channel variables of food preference and dietary knowledge, we use responses of the CHNS for respondents over 12 years that were collected since 2004. As described in Section 2.1, we considered individuals aged 18–65 at the time of the survey. Based on five questions concerning consumers’ preference for food consumption, the respondents are asked to indicate their preference for each food item, including fast food, salty snack foods, fruits, vegetables, soft drinks, and sugared fruit drinks. A final score is calculated based on the respondents’ answers; a value of 1 indicates a healthy preference, a value of −1 indicates an unhealthy preference, and 0 represents a “neutral” preference. The higher the score, the healthier the preference (Supplementary Table S2). This shows that females have a significantly higher preference score compared to males, as shown in Table 1.

Dietary knowledge scores are calculated based on five questions on basic dietary knowledge (Supplementary Table S3). Referring to the standard suggested by the World Health Organization [43] (WHO), we constructed a brief index according to the respondents’ answers: 1 for a correct answer, −1 for an incorrect answer, and 0 for an “unknown” answer. This measurement method has been widely used to evaluate individuals’ dietary knowledge [19,38]. The higher the score, the greater the knowledge of nutritional intake. Females seem to have a slightly lower score in dietary knowledge than males, but the difference is not statistically significant considering a significance level of 0.05 (Table 1).

Following previous studies of the estimation of nutrition [19,23], we also controlled for individual demographic variables (gender, ethnicity, age, age squared, marital status, education, and working status, in addition to risk behaviors of smoking and drinking, heavy activity) and household characteristics (residence, household size, logarithm of income). Provincial dummies were used to control for province-specific and time-invariant characteristics, for instance, for cultural or geographic features that were unchanged during the survey period considered. Time dummies are used to control for time fixed effects, such as nationwide policies or economic shocks that can vary during survey years but have an equal influence on individuals across provinces. The details regarding the statistics of the control variables are presented in Table 1.

### 2.3. Method

The econometric model used in this study is defined as follows:

(1) $${Diet}_{it}=\alpha_{1}{Urbanization}_{it}+\alpha_{2}{Urbanization}_{it}^{2}+\beta_{0}X_{it}+\gamma_{0}Z_{i}+\xi_{it}.$$

${Diet}_{it}$ represents the variables that reflect the dietary transition, including the calorie intakes and dietary quality. ${Urbanization}_{it}$ is our main explanatory variable, and it is constructed as a comprehensive index (the details regarding the urbanization index are given in Section 2.2). To examine whether a nonlinear relationship exists between urbanization and dietary transition, we introduce the quadratic term of urbanization in the estimation. $X_{it}$ is the vector for the control variables. $Z_{i}$ is used to control for individual fixed effects; it is assumed to be time invariant. $\varepsilon_{i}$ is the disturbance and assumed to be $\xi_{it}\sim N\left(0,\;1\right)$.

Since our data have a panel structure, the conventional procedure is to follow a standard panel estimation method by estimating random effects (RE), fixed effects (FE), or the first difference (FD) for Equation (1) and to conduct the Hausmann test to check which model is appropriate. However, in our case, key variables for measuring dietary transition have little variation over the survey years, implying that FE or FD methods might lead to imprecise estimates, because many observations would be dropped from the estimation [44]. Following the method developed by Mundlak [45] and widely discussed and used by other researchers [19,44,46,47], we apply a pseudo-fixed-effects estimator, the Mundlak estimator, as an additional comparison to the RE estimates. The main advantage of the Mundlak (MK) estimator is that it can control for bias that may arise from individual heterogeneity and omitted time-varying variables [44,48] by including covariate mean values as additional explanatory variables in the estimation. In this way, the individual heterogeneity can be addressed with the MK estimator if the joint significance test of the mean value of all time-varying covariates is statistically significant.

## 3. Results

### 3.1. The Effect of Urbanization on Dietary Transition

To investigate how urbanization affects the dietary transition, we conducted the estimations nutrition intake (intake of calories and the three macronutrients) and the HEI, respectively. For a straightforward interpretation, the urbanization index was standardized, and the logarithm of nutrition intakes was used in the estimations. The estimates from the RE estimator and MK estimator are presented in Table 2, Table 3 and Table 4. In general, the results from the MK estimator are largely consistent with the results from the RE estimator, and the joint test of the Mundlak mean implies that individual fixed effects are to be considered. Thus, the MK estimator is preferred. The interpretation rests on the estimates from the MK estimation.

As shown in columns (1) and (2) in Table 2 from the RE and MK estimations, urbanization has statistically significant and negative effects on calorie intake, with the estimate of urbanization from the MK estimation being slightly lower. Following the joint test results that the MK estimator is the preferred estimator, a one standard deviation increase in urbanization reduces the calorie intake by 1.%. This suggests that with increasing urbanization, rural residents have a lower calorie intake. To investigate the nonlinear relationship between urbanization and calorie intake, we introduced the quadratic term of urbanization in the estimation as shown in columns (3) and (4). However, the coefficient of urbanization squared is insignificant, suggesting that no quadratic relationship exists between urbanization and calorie intake.

To measure how urbanization affects dietary transition, the estimates for the effect of urbanization on each macronutrient are presented in Table 3. As shown in Table 3, the results from RE and MK estimations are largely consistent. Specifically, the estimations for carbohydrates show results similar to those for calorie intake: urbanization has a statistically significant and negative effect on carbohydrate consumption; the insignificant coefficient of urbanization squared indicates the effect is monotone. The results suggest that increasing urbanization consistently reduces the consumption of carbohydrates, reflecting the change in the food consumption of rural residents from staple foods, which are characterized as cereal products, to other sources of nutrients [34]. This argument is further supported by the results from the estimations for fat and protein, which both show statistically significant and positive relationships with urbanization. However, the coefficient of urbanization squared in the estimation for fat intake is statistically significant and negative, suggesting an inverted U-shaped relationship between urbanization and fat intake [23]. By comparison, the significantly positive coefficient of urbanization squared in the estimation for protein intake supports a U-shaped relationship between urbanization and protein intake.

The estimations for the effect of urbanization on the percentage contributions of the three macronutrients are presented in Table 4. In general, similar patterns are found for the percentage contributions of the three macronutrients. The percentage contribution of carbohydrates in total calorie intake tends to consistently decrease with increasing urbanization, whereas the percentage contribution of fat indicates an increasing trend with increasing urbanization. There is a U-shaped relationship between the percentage contribution of protein and urbanization, indicating that increasing urbanization first drives a decrease and then an increase in the percentage contribution of protein after a certain level of urbanization.

**Figure 2:**
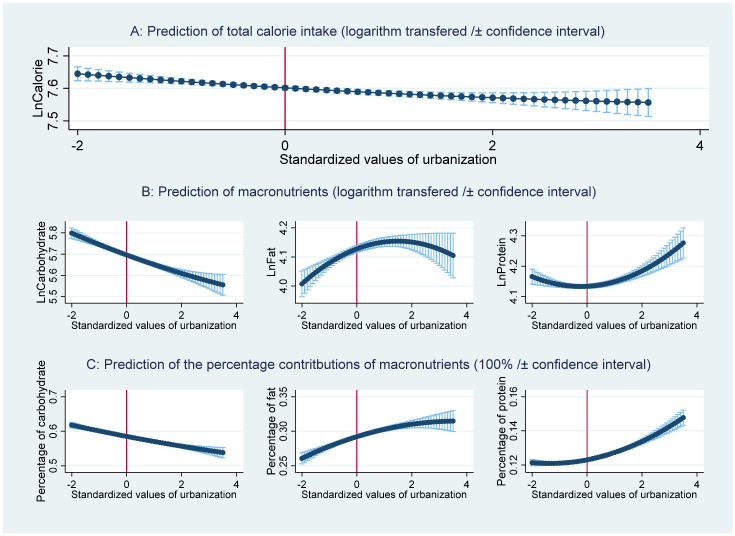
The relationship between urbanization and the predicted nutrition intakes and their percentage contributions.

The relationships between urbanization and the predicted value of calorie intake and the three macronutrients are presented in Figure 2. It shows a monotonically decreasing relationship between calorie intake and carbohydrate consumption with increasing urbanization. The inverted U-shaped relationship between urbanization and fat intake reveals that fat consumption first increases and then decreases after the turning point (standardized urbanization index = −(0.036/(2 × (−0.012))) = 1.5, or urbanization index = 1.5 × 16.11 + 56.00 = 80.17). For protein intake, the marginal effect shows a slightly U-shaped relationship between urbanization and protein intake, suggesting that protein consumption first decreases and then increases after the turning point (standardized urbanization = −(0.005/(2 × 0.010)) = −0.25, or urbanization = −0.25 × 16.11 + 56.00 = 51.97). Interestingly, the percentage contribution of fat shows an increasing trend, but the increasing trend tends to decrease with increasing urbanization. To summarize, accelerating urbanization tends to reduce calorie intake, which consists of fewer carbohydrates and fewer fats when urbanization is equal to or exceeds the threshold of 80.17, but greater protein consumption when urbanization is equal to or exceeds the threshold of 51.97.

**Figure 3:**
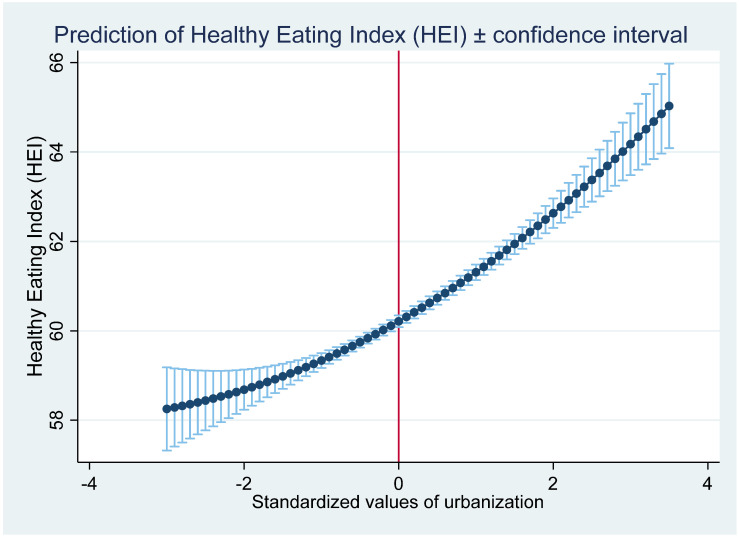
The relationship between urbanization and the predicted Healthy Eating Index (HEI).

Since the nutrition intakes can only be used to examine the quantity and patterns of dietary change, the HEI can be used to investigate how urbanization affects the dietary quality. As shown in Table 5, column (2) from the MK estimation, urbanization shows a significant and positive effect on the HEI. The estimation in column (4) with the inclusion of urbanization squared indicates that a U-shaped relationship exists between urbanization and the HEI. However, the relationship between the predicted HEI and urbanization, as shown in Figure 3, supports a monotonically increasing trend. The reason is that all of the observations in our estimation are located at the right side of the U-shape. Thus, we conclude that dietary quality tends to improve with increasing urbanization.

### 3.2. Robustness Check

**Figure 4:**
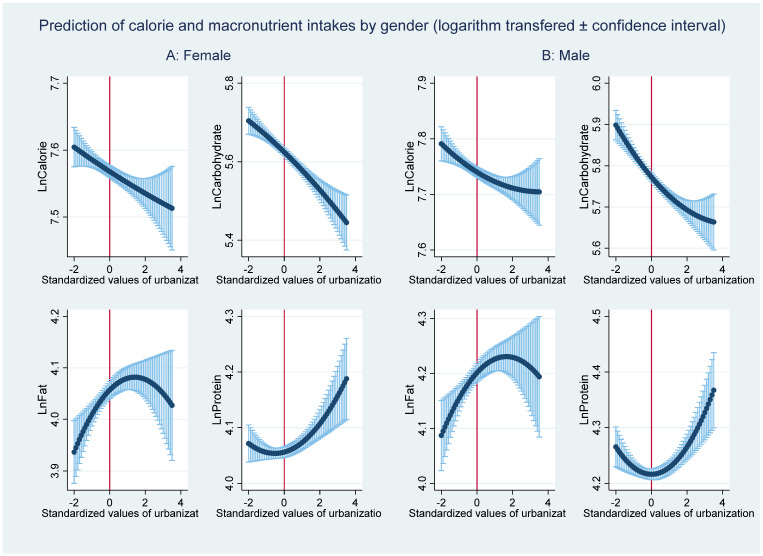
The relationship between urbanization and the predicted nutrition intakes by gender.

**Figure 5:**
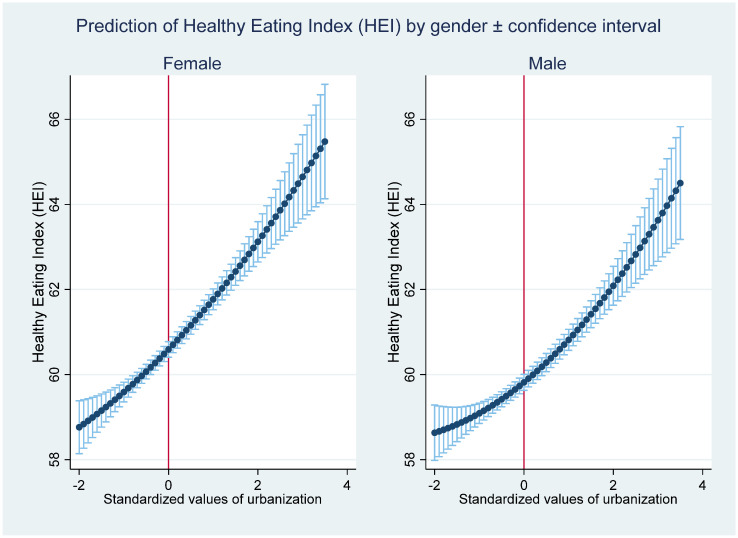
The relationship between urbanization and the predicted Healthy Eating Index (HEI) by gender.

To check whether our results hold for different subsamples, we conducted estimations for male and female samples (Table 6). Regarding the pooled samples, urbanization has a statistically significant and negative effect on calorie and carbohydrate intakes for the male and female samples. The urbanization effect tends to be slightly smaller for females than for males. Figure 4 and Figure 5 show that the main patterns of the relationships between urbanization and predicted dietary intakes for females and males are strongly consistent with the patterns for the pooled sample. The fat intake shows an inverted U-shape, and the protein intake shows a U-shape with increasing urbanization. The turning points for females are lower than are those for males; this indicates that females tend to change their dietary patterns earlier in the process of urbanization. In line with previous studies, females are more likely to adjust their behavior toward better nutrition-related health [49]. Moreover, regarding the effect of urbanization on the HEI, urbanization has a higher effect for females than it has for males. This suggests that females become more aware of dietary quality with increasing urbanization than males.

### 3.3. The Effect of Urbanization on Food Preference and Dietary Knowledge

**Figure 6:**
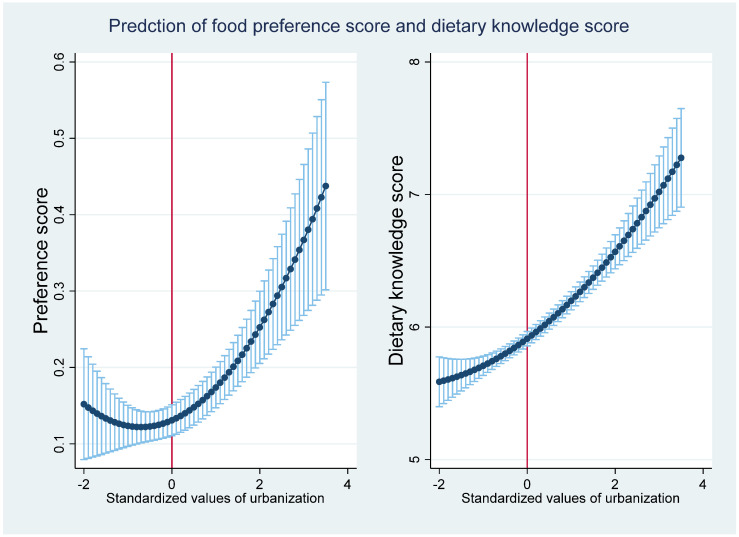
The relationship between urbanization and the predicted food preference and dietary knowledge.

To better understand how urbanization affects dietary transition, we estimated the effect of urbanization on food preference and dietary knowledge (Table 7). The results indicate that there exists a U-shaped relationship between urbanization and food preferences and dietary knowledge. The turning point is calculated as follows: standardized urbanization index = −(0.025/(2 × 0.017)) = −0.74 or urbanization index = −0.74 × 16.11 + 56.00 = 67.85. The relationship between the urbanization index and the predicted values of food preference and dietary knowledge is presented in Figure 6. Figure 6 shows that the food preference score first increases and then decreases after the estimated turning point, whereas the dietary knowledge score shows a monotonically increasing trend with urbanization, indicating that all of our observations are located at the right side of the U-shape. With increasing urbanization, healthier food preferences (after the urbanization index threshold of 67.85) and better dietary knowledge emerge (Figure 6).

## 4. Discussion

Since the level of urbanization in China approximately trebled from 1979 to 2018, China has experienced rapidly changing dietary patterns and the co-existence of overweight and obesity, undernutrition, and micronutrient deficiencies, especially in rural areas [6,50]. China’s government continues to promote urbanization in rural areas with mixed consequences for the food environment and in the socioeconomic, cultural, and environmental domains.

In rural China, the food infrastructure has been largely improved, such as through the construction of modern food markets that could have significantly influenced the food availability and nutrition status of the local residents [4,20,23]. In addition, increasing urbanization has changed rural residents’ lifestyles in several ways—for example, a change toward more sedentary job choices and lifestyles that require lower calorie consumption [16]. This development has possibly resulted in a decreasing trend of daily calorie intake. The underlying causes could be linked to a conscious decision of dietary shift or could be naturally occurring or both. Increased food prices from urbanization could also be a potential reason for this trend, but studies have shown that the nutritional impact of food prices is small for poor households in China. First, households can substitute to cheaper foods and second, the government has strict interventions in grain markets to keep domestic prices of staple foods low [51].

Against this background, our findings suggest that with increasing urbanization, the quantity and percentage contribution of carbohydrate consumption has decreased considerably in China. The composition of macronutrients has shifted from carbohydrate-based diets toward dietary patterns that are oriented toward higher amounts of fat and protein [34]. Fat consumption shows a decreasing trend after a certain level of urbanization, in line with previous studies [23], whereas we find that its percentage contribution to total calories continues to show an increasing trend. In contrast, the intake of protein shows an increasing trend after a certain level of urbanization. Our results also show that with increasing urbanization, rural residents are more likely to have healthier food preferences and better dietary knowledge, which in turn might have a positive effect on their nutrition patterns, thereby offsetting some of the pressure on the food system caused by a higher demand for animal proteins. There might also exist an interplay between dietary knowledge and food consumption preference. For instance, dietary knowledge tends to affect dietary quality such as through lowering total calorie intake, and this effect is larger for overweight adults [38]. This suggests that improving the dietary knowledge of rural residents might play an important role in shifting less healthy rural residents toward a more healthful diet. However, it should be noted that higher dietary knowledge does not necessarily suggest a healthy diet, as there are also other factors determining rural residents’ food consumption behaviors such as the availability of healthy food products and their health consciousness.

Regarding international comparison, Chinese diets are still characterized by relatively high levels of carbohydrates, whereas the consumption of animal proteins, such as meat, animal fats and oils, and dairy products, is traditionally lower compared to developed economies or other transitional economies, such as Russia [52]. This trend also suggests that the demand for protein from animal sources will possibly increase in the future, thereby putting additional pressure on the food system and the environment and possibly contributing to climate change. Previous studies have clearly documented that animal products have the highest income (urbanization) elasticities and, due to increasing income (urbanization), the demand for animal products will possibly increase considerably [11,12]. Thus, the urbanization of rural areas could put pressure on agricultural land and, consequently, the agricultural workforce, and it could trigger land conflicts [53].

Another aspect is the increasing numbers of rural residents that are migrating to urban areas for work, accounting for roughly 20% of the national total population in 2018. From the perspective of nutrition transition, people living in developed regions tend to have healthier food consumption behaviors and nutrition intakes; in addition, they are more likely to change their unhealthy consumption behaviors earlier [49,54]. Rural residents who are non-migrants and remain in rural areas usually experience nutrition transition because of economic development or urbanization. In contrast, rural migrants who leave the rural area to work in an urban area are largely affected by the food consumption patterns of urban residents and are likely to follow the urban residents’ food consumption behaviors and could acquire knowledge of healthy nutrition [55]. Nevertheless, in urban areas, there might also be more fast-food options, alcohol consumption among peers, poor living arrangements, etc. [4], which cannot be overlooked in the nutrition transition of rural migrants. Rural migrants could also experience a healthier food consumption style, such as eating more vegetables or fruits instead of mainly staple foods, compared to rural residents in their hometown [56]. Rural migrants’ healthier food consumption styles might then positively influence their families at home, leading to an indirect health benefit [57]. These arguments support the claim that rural migrants might play a significant role in the nutrition transition in rural areas. This could accelerate positive change in rural residents’ food consumption patterns and help them adopt a healthier diet in the process of nutrition transition through positive peer effects and learning in urban areas. Negative social consequences from migration to urban areas cannot be overlooked but are out of the scope of this article.

There are several limitations in our study. First, our data is from the CHNS which might be “unique” to China, as Chinese diets are still characterized by relatively high levels of carbohydrates, but the consumption of animal proteins, such as meat, animal fats and oils, and dairy products, is traditionally lower compared to developed economies or other transitional economies, such as Russia [52]. Second, the complex quality differences in the macronutrients of carbohydrates, fats, and proteins that influence nutrition quality are not considered in this study due to data limitation. However, this is important for defining a healthy diet. For instance, saturated fat is considered unhealthy, but mono-or poly-unsaturated fat is argued to be healthy [58]. Third, the CHNS used three-day food diaries, which might under/over report the real quantity of food consumed, although it has been corrected by using a food inventory taken at the household level over the same three-day period. Finally, our results should be interpreted for the survey period from 2004 to 2011, considering that China has experienced significant changes in urbanization and nutrition transition in recent years. Thus, this study is a first attempt for better understanding the nutrition transition resulting from accelerating urbanization in rural China. Further investigation of the quality differences in the macronutrients is crucial for fully understanding the dietary transition in rural China through urbanization. The nexus of the complex interactions between urbanization, dietary transition, the society, and the environment requires further attention in future studies.

## 5. Conclusions

Using the CHNS data, we find that increasing urbanization tends to lower the dietary intake (measured by the intakes of carbohydrate, fat, and protein) of rural residents, but it increases their dietary quality (measured with the HEI [40]) for the considered survey period from 2004 to 2011. The structure of the macronutrients, food preferences, and dietary knowledge seem to be crucial in explaining the dietary transition in rural China. Specifically, in rural China, our findings suggest that from 2004 to 2011 increasing urbanization consistently reduces carbohydrate consumption, decreases fat consumption after the urbanization turning point, and increases protein consumption after the turning point. Increased urbanization is also found to positively associate with rural residents’ healthy food preferences and their dietary knowledge in the period considered.

## Figures and Tables

**Table 1:** Descriptive statistics of the main variables.

		Pooled	Females	Males	Diff (3)–(2) ^a^
		(1)	(2)	(3)	(4)
Dependent Variables					
Calories	Average daily calorie intake in three days (kcal)	2215.50	2029.46	2412.08	382.62 **
		(685.92)	(611.22)	(705.48)	
Carbohydrate	Average daily carbohydrate intake in three days (g)	319.58	296.19	344.29	48.09 **
		(115.69)	(107.05)	(119.29)	
Fat	Average daily fat intake in three days (g)	71.11	65.98	76.52	10.54 **
		(37.15)	(33.92)	(39.57)	
Protein	Average protein intake in three days (g)	67.02	61.70	72.65	10.95 **
		(23.54)	(21.29)	(24.49)	
Percentage of Carbohydrate	Percentage contribution of carbohydrate to total calorie intake	0.59	0.59	0.59	0.00
		(0.12)	(0.12)	(0.12)	
Percentage of Fat	Percentage contribution of fat to total calorie intake	0.29	0.29	0.29	−0.01 ** ^b^
		(0.11)	(0.11)	(0.11)	
Percentage of Protein	Percentage contribution of protein to total calorie intake	0.12	0.12	0.12	−0.00 ** ^c^
		(0.03)	(0.03)	(0.03)	
HEI	Healthy Eating Index for Chinese	60.33	60.66	59.98	−0.68 **
		(6.83)	(6.82)	(6.83)	
Independent Variables					
Urbanization	Urbanization Index	56.00	55.76	56.25	0.49
		(16.11)	(16.08)	(16.15)	
Channel Variables					
Preference score	Score of healthy food consumption preference	0.15	0.19	0.11	−0.07 **
		(1.00)	(0.98)	(1.01)	
Knowledge score	Score of dietary knowledge	5.95	5.90	6.01	0.12
		(3.65)	(3.67)	(3.62)	
Number of groups		8023	4025	3998	
Observations		17,315	8896	8419	

Notes: The mean values are presented and standard deviations are in parentheses; ** *p* < 0.01. ^a^: *t*-tests are applied. ^b^: the difference in the percentage contribution of fats between males and females is 0.007. ^c^: the difference in the percentage contribution of proteins between males and females is 0.003.

**Table 2:** The effect of urbanization on calorie intake.

Variables	Dependent Variable: LnCalorie ^a^			
	(1)	(2)	(3)	(4)
	RE ^b^	MK ^c^	RE	MK
Urbanization	−0.022 **	−0.017 **	−0.023 **	−0.018 **
	(0.00)	(0.00)	(0.00)	(0.00)
Urbanization squared			0.001	0.002
			(0.00)	(0.00)
Controls	Yes	Yes	Yes	Yes
MK mean	No	Yes	No	Yes
Wald chi2	3894 **	4021 **	3907 **	4037 **
Joint test for MK mean		86.0 **		86.3 **
Number of observations	17,315	17,315	17,315	17,315
Number of individuals	8023	8023	8023	8023

Notes: Standard errors are in parentheses; ** *p* < 0.01. ^a^: The dependent variable of LnCalorie is the logarithm of Calories. ^b^: RE refers to random effect estimation. ^c^: MK refers to Mundlak (MK) estimation.

**Table 3:** The effect of urbanization on the intake of macronutrients.

Variables	LnCarbohydrate ^c^		LnFat		LnProtein	
	(1)	(2)	(3)	(4)	(5)	(6)
Panel A: RE estimation ^a^						
Urbanization	−0.058 **	−0.058 **	0.038 **	0.045 **	0.010 **	0.003
	(0.00)	(0.00)	(0.01)	(0.01)	(0.00)	(0.00)
Urbanization squared		0.000		−0.009 *		0.011 **
		(0.00)		(0.00)		(0.00)
Controls	Yes	Yes	Yes	Yes	Yes	Yes
Wald chi2	5401 **	5419 **	2032 **	2036 **	3136 **	3138 **
Number of observations	17,315	17,315	17,311	17,311	17,315	17,315
Number of individuals	8023	8023	8020	8020	8023	8023
Panel B: MK estimation ^b^						
Urbanization	−0.046 **	−0.047 **	0.028 **	0.036 **	0.011 **	0.005
	(0.00)	(0.00)	(0.01)	(0.01)	(0.00)	(0.00)
Urbanization squared		0.002		−0.012 **		0.010 **
		(0.00)		(0.00)		(0.00)
Controls	Yes	Yes	Yes	Yes	Yes	Yes
MK mean	Yes	Yes	Yes	Yes	Yes	Yes
Wald chi2	5670 **	5702 **	2138 **	2144 **	3134 **	3135 **
Joint test for MK mean	167.6 **	168.4 **	84.2 **	87.3 **	79.2 **	78.1 **
Number of observations	17,315	17,315	17,311	17,311	17,315	17,315
Number of individuals	8023	8023	8020	8020	8023	8023

Notes: Standard errors are in parentheses; ** *p* < 0.01; * *p* < 0.05. ^a^: RE refers to random effect estimation. ^b^: MK refers to Mundlak (MK) estimation. ^c^: The dependent variables of LnCarbohydrate, LnFat, and LnProtein are the logarithms of calories, carbohydrate, cat, and protein intakes.

**Table 4:** The effect of urbanization on the percentage contributions of the macronutrients.

Variables	Percentage of Carbohydrate		Percentage of Fat		Percentage of Protein	
	(1)	(2)	(3)	(4)	(5)	(6)
Panel A: RE estimation ^a^						
Urbanization	−0.020 **	−0.020 **	0.015 **	0.015 **	0.004 **	0.003 **
	(0.00)	(0.00)	(0.00)	(0.00)	(0.00)	(0.00)
Urbanization squared		0.000		−0.000		0.001 **
		(0.00)		(0.00)		(0.00)
Controls	Yes	Yes	Yes	Yes	Yes	Yes
Wald chi2	5120 **	5160 **	2883 **	2911 **	4009 **	4022 **
Number of observations	17,315	17,315	17,315	17,315	17,315	17,315
Number of individuals	8023	8023	8023	8023	8023	8023
Panel B: MK estimation ^b^						
Urbanization	−0.016 **	−0.016 **	0.011 **	0.012 **	0.004 **	0.003 **
	(0.00)	(0.00)	(0.00)	(0.00)	(0.00)	(0.00)
Urbanization squared		0.001		−0.001		0.001 **
		(0.00)		(0.00)		(0.00)
Controls	Yes	Yes	Yes	Yes	Yes	Yes
MK mean	Yes	Yes	Yes	Yes	Yes	Yes
Wald chi2	5342 **	5393 **	3045 **	3087 **	4173 **	4187 **
Joint test for MK mean	234.8 **	230.6 **	159.0 **	160.7 **	124.0 **	112.3 **
Number of observations	17,315	17,315	17,315	17,315	17,315	17,315
Number of individuals	8023	8023	8023	8023	8023	8023

Notes: Standard errors are in parentheses; ** *p* < 0.01. ^a^: RE refers to random effect estimation. ^b^: MK refers to Mundlak (MK) estimation.

**Table 5:** The effect of urbanization on the Healthy Eating Index (HEI).

Variables	Dependent Variable: Healthy Eating Index (HEI)			
	(1)	(2)	(3)	(4)
	RE ^a^	MK ^b^	RE	MK
Urbanization	1.132 **	1.058 **	1.047 **	0.987 **
	(0.06)	(0.07)	(0.07)	(0.07)
Urbanization squared			0.128 **	0.111 *
			(0.04)	(0.04)
Controls	Yes	Yes	Yes	Yes
MK mean	No	Yes	No	Yes
Wald chi2	3707 **	3777 **	3712 **	3780 **
Joint test for MK mean		71.59 **		69.54 **
Observations	17,315	17,315	17,315	17,315
Individuals	8023	8023	8023	8023

Notes: Standard errors are in parentheses; ** *p* < 0.01; * *p* < 0.05. ^a^: RE refers to random effect estimation. ^b^: MK refers to Mundlak (MK) estimation.

**Table 6:** The effect of urbanization on nutrition and the Healthy Eating Index (HEI) by gender.

Variables ^a^	LnCalories		LnCarbohydrate		LnFat		LnProtein		HEI	
	(1)	(2)	(3)	(4)	(5)	(6)	(7)	(8)	(9)	(10)
Panel A: Female sample										
Urbanization	−0.017 **	−0.017 **	−0.045 **	−0.044 **	0.028 **	0.035 **	0.014 **	0.009	1.144 **	1.090 **
	(0.00)	(0.00)	(0.01)	(0.01)	(0.01)	(0.01)	(0.01)	(0.01)	(0.09)	(0.10)
Urbanization squared		0.000		−0.002		−0.012 *		0.008 *		0.087
		(0.00)		(0.00)		(0.01)		(0.00)		(0.06)
Controls	Yes	Yes	Yes	Yes	Yes	Yes	Yes	Yes	Yes	Yes
MK mean ^b^	Yes	Yes	Yes	Yes	Yes	Yes	Yes	Yes	Yes	Yes
Wald Chi2	1465 **	1472 **	2676 **	2687 **	1038 **	1042 **	1064 **	1063 **	2044 **	2045 **
Joint test for MK mean ^b^	47.1 **	47.1 **	92.6 **	91.9 **	53.5 **	54.9 **	33.8 **	33.3 **	44.4 **	43.1 **
Observations	8896	8896	8896	8896	8896	8896	8896	8896	8896	8896
Individuals	4025	4025	4025	4025	4025	4025	4025	4025	4025	4025
Panel B: Male sample										
Urbanization	−0.018 **	−0.020 **	−0.048 **	−0.051 **	0.029 **	0.036 **	0.008 *	−0.000	0.953 **	0.864 **
	(0.00)	(0.00)	(0.01)	(0.01)	(0.01)	(0.01)	(0.00)	(0.01)	(0.09)	(0.10)
Urbanization squared		0.003		0.006		−0.011		0.012 **		0.136 *
		(0.00)		(0.00)		(0.01)		(0.00)		(0.06)
Controls	Yes	Yes	Yes	Yes	Yes	Yes	Yes	Yes	Yes	Yes
MK mean ^b^	Yes	Yes	Yes	Yes	Yes	Yes	Yes	Yes	Yes	Yes
Wald Chi2	1266 **	1271 **	2309 **	2329 **	939 **	942 **	1114 **	1114 **	1764 **	1765 **
Joint test for MK mean ^b^	42.8 **	43.1 **	73.1 **	74.8 **	42.1 **	43.3 **	48.5 **	48.0 **	47.7 **	47.0 **
Observations	8419	8419	8419	8419	8419	8419	8419	8419	8419	8419
Individuals	3998	3998	3998	3998	3998	3998	3998	3998	3998	3998

Notes: Standard errors are in parentheses; ** *p* < 0.01; * *p* < 0.05. ^a^: The dependent variables of LnCalories, LnCarbohydrate, LnFat, and LnProtein are the logarithm of calories, carbohydrate, fat, and protein intakes; ^b^: MK refers to Mundlak (MK) estimation.

**Table 7:** The effect of urbanization on food preference and dietary knowledge.

Variables	Preference Score		Knowledge Score	
	(1)	(2)	(3)	(4)
Urbanization	0.036 **	0.025 *	0.272 **	0.245 **
	(0.01)	(0.01)	(0.03)	(0.03)
Urbanization squared		0.017 **		0.041 *
		(0.01)		(0.02)
Controls	Yes	Yes	Yes	Yes
MK mean ^a^	Yes	Yes	Yes	Yes
Wald chi2	307 **	318 **	24,592 **	24,710 **
Joint test for MK mean ^a^	33.5 **	33.4 **	27.9 **	27.1 **
Observations	17,315	17,315	17,315	17,315
Individuals	8023	8023	8023	8023

Notes: Standard errors are in parentheses; ** *p* < 0.01; * *p* < 0.05. ^a^: MK refers to Mundlak (MK) estimation.

## Data Availability

The raw data used in this research can be downloaded directly from the China Health and Nutrition Survey (https://www.cpc.unc.edu/projects/china/data/datasets, accessed on 12 October 2020).

## Supplementary Materials

The following supplementary tables are available online at https://www.mdpi.com/2072-6643/13/3/921/s1, Table S1: Recommended amounts of food groups, expressed in grams; Table S2: Questions concerning dietary knowledge in the CHNS; Table S3: Questions concerning food preference in the CHNS.
