# Supplementary material for: Nutrition Transition with Accelerating Urbanization? Empirical Evidence from Rural China
Source: Nutrients. 2021 Mar 12;13(3):921. doi: 10.3390/nu13030921 (PMC7999076; doi:10.3390/nu13030921)
Supplement: Supplementary file 1 [file nutrients-13-00921-s001.pdf]

Supplement:

Table S1. Recommended amounts of food groups (Grams)

**Food Group (g)**	**Weights** [3]	**Calorie level (kcal)**										
		1000	1200	1400	1600 [4]	1800	2000	2200	2400	2600	2800	3000
Adequacy [1]												
Total Grains	5	85	100	150	200	225	250	275	300	350	375	400
Whole Grains and Mixed Beans	5				50	75	100	125	150			
Tubers	5				50	50	75	75	100	125	125	125
Vegetables	5	200	250	300	300	400	450	450	500	500	500	600
Dark Vegetables	5	100	125	150	150	200	225	225	250	250	250	300
Fruits	10	150	150	150	200	200	300	300	350	350	400	400
Dairy	5	500	500	350	300	300	300	300	300	300	300	300
Soybeans	5	5	15	15	15	15	15	25	25	25	25	25
Seeds and Nuts	5				10	10	10	10	10	10	10	10
Fish and Seafood	5	15	20	40	40	50	50	75	75	75	100	125
Poultry	5	15	25	40	40	50	50	75	75	75	100	100
Eggs	5	20	25	25	40	40	50	50	50	50	50	50
Limitation [2]												
Red Meat	5	Score = 0 if amounts ≥ 260 g; score = 10 if amounts ≤ 30 g										
Cooking Oils	10	Score = 0 if amounts ≥ 52.16 g; score = 10 if amounts ≤ 25 g										
Sodium	10	Score = 0 if amounts ≥ 5772 mg; score = 10 if amounts ≤ 1600 mg										
Added Sugars	5	Score = 0 if amounts ≥ 20 % of energy; score = 5 if amounts ≤ 10 % of energy										
Alcohol	5	Score = 0 if amounts ≥ 25 g (men) / 15 g (women); score = 5 if amounts ≤ 60 g (men) / 40 g (women)										

Notes:

[1] Foods are divided into two groups: Adequacy Group and Limitation Group. In the adequacy group, if daily intakes are more than the recommended value, it will get a score of the total weight. If daily intakes equal to 0, the score will be 0. The weighted score is used when daily intakes are ranging between 0 and the recommended value.

[2] In the limitation group, if daily intakes are less than the recommended value, it will get a score of the total weight. If daily intakes are more than the upper value, the score will be 0. The weighted score is used when daily intakes are ranging between 0 and the recommended value.

[3] The weight of each food item is provided in this column.

[4] We take the recommended amounts on the calorie level of 1600 kcal.

Sources: Yuan, Y.-Q., Li, F., Dong, R.-H., Chen, J.-S., He, G.-S., Li, S.-G., & Chen, B. (2017). The Development of a Chinese Healthy Eating Index and Its Application in the General Population. *Nutrients*, *9*(9).

Table S2. Questions concerning dietary knowledge in the CHNS.

Dietary knowledge:	
Do you strongly agree, somewhat agree, are neutral, somewhat disagree or strongly disagree with this statement?	True/ False
**Please note that the question is not asking about your actual habits.*	
Q1: Choosing a diet with a lot of fresh fruit and vegetables is good for one's health	T
Q2: Eating a lot of sugar is good for one's health	F
Q3: Eating a variety of foods is good for one's health	T
Q4: Choosing a diet high in fat is good for one's health	F
Q5: Choosing a diet with a lot of staple foods (rice and rice products and wheat and wheat products) is not good for one's health	T
Q6: Consuming a lot of animal products daily (fish, poultry, egg and lean meat) is good for one's health	F
Q7: Reducing the amount of fatty meat and animal fat in the diet is good for one's health	T
Q8: Consuming milk and dairy products is good for one's health	T
Q9: Consuming beans and bean products is good for one's health	T
Q10: Physical activities are good for one's health	T
Q11: Sweaty sports or other intense physical activities are not good for one's health	T
Q12: The heavier one's body is, the healthier he or she is	F
Index rules: "1" point was given for a correct answer, "-1" point for an incorrect answer, and "0" points for the other answers.	

Source: The dietary knowledge questionnaire is from the official website of the China Health and Nutrition Survey. (http://www.cpc.unc.edu/projects/china)

Table S3. Questions concerning food preference in the CHNS.

Food Preference:	
How much do you like this food: Like very much, like somewhat, neutral, dislike somewhat, or dislike very much?	Healthy (H)/ Unhealthy (U)
Q1: Fast food (KFC, pizza, hamburgers, etc.)	U
Q2: Salty snack foods (potato chips, pretzels, French fries, etc.)	U
Q3: Fruits	H
Q4: Vegetables	H
Q5: Soft drinks and sugared fruit drinks	U
Index rules: "1" point was given for liking a healthy preference, "-1" point for liking an unhealthy preference, and "0" points for neutral.	

Source: The dietary knowledge questionnaire is from the official website of the China Health and Nutrition Survey. (http://www.cpc.unc.edu/projects/china)
